# Daily urinary urea excretion to guide intermittent hemodialysis weaning in critically ill patients

**DOI:** 10.1186/s13054-016-1225-5

**Published:** 2016-02-19

**Authors:** Julien Aniort, Ali Ait Hssain, Bruno Pereira, Elisabeth Coupez, Pierre Antoine Pioche, Christophe Leroy, Anne Elisabeth Heng, Bertrand Souweine, Alexandre Lautrette

**Affiliations:** Nephrology, Dialysis and Transplantation Department, Gabriel Montpied Teaching Hospital, University Hospital of Clermont-Ferrand, Clermont-Ferrand, France; Medical Intensive Care Unit, Gabriel Montpied Teaching Hospital, University Hospital of Clermont-Ferrand, 54 rue Montalembert, BP69, 63003 Clermont-Ferrand, Cedex 1 France; Biostatistics and Research Department (DRCI), University Hospital of Clermont-Ferrand, Clermont-Ferrand, France; Laboratoire Micro-organismes: Génome et Environnement (LMGE), UMR CNRS 6023, Clermont-University, Clermont-Ferrand, France

**Keywords:** Acute kidney injury, Renal replacement therapy, Intermittent hemodialysis, Weaning, Critical illness, Intensive care

## Abstract

**Background:**

There are no easily available markers of renal recovery to guide intermittent hemodialysis (IHD) weaning. The aim of this study was to identify markers for IHD weaning in critically ill patients with acute kidney injury (AKI).

**Methods:**

We performed a retrospective single-center cohort study of patients treated with IHD for at least 7 days and four dialysis sessions for AKI between 2006 and 2011 in an intensive care unit (ICU) of a French university hospital. Blood and urinary markers were recorded on the day of the last IHD in the ICU for unweaned patients and 2 days after the last IHD for weaned patients. Factors associated with IHD weaning were identified by multiple logistic regression. The areas under the receiver operating characteristic curve (AUROC) and the characteristics of the best diagnostic thresholds were compared.

**Results:**

Sixty-seven patients were analyzed, including thirty-seven IHD-weaned patients. Urine output [odds ratio (OR) 1.59, 95 % confidence interval (CI) 1.20–2.10 (per ml/kg/24 h increase); *P* = 0.01] and urinary urea concentration [OR 1.29, 95 % CI 1.01–1.64 (per 10 mmol/L increase); *P* = 0.04] were both associated with IHD weaning. The optimal diagnostic thresholds for IHD weaning were urine output greater than 8.5 ml/kg/24 h, urinary urea concentration greater than 148 mmol/L, and daily urea excretion greater than 1.35 mmol/kg/24 h, with accuracy of 82.1 %, 76.1 %, and 92.5 % (*P* = 0.03), respectively. The AUROC of daily urinary urea excretion (0.96) was greater than the AUROC of urine output (0.86) or the AUROC of urinary urea concentration (0.83) (*P* < 0.001).

**Conclusions:**

A daily urinary urea excretion greater than 1.35 mmol/kg/24 h was found to be the best marker for weaning ICU patients with AKI from IHD.

**Electronic supplementary material:**

The online version of this article (doi:10.1186/s13054-016-1225-5) contains supplementary material, which is available to authorized users.

## Background

In intensive care units (ICUs), renal replacement therapy (RRT) for acute kidney injury (AKI) increases the risk of bleeding, hemodynamic instability, underdosage of antibiotics, nutrient loss, and infections [1–5]. These complications increase with the length of RRT and lead to increased mortality and decreased likelihood of renal recovery [6]. Improving RRT modalities has had a positive effect on the outcomes of ICU patients with AKI [7–11]. The length of RRT has an impact on mortality and renal recovery [12]. Most studies have focused on the timing of RRT initiation [13–15], and very few have assessed the criteria for RRT weaning. Although researchers in one study reported that creatinine clearance could help in making decisions about when to discontinue continuous RRT, serum creatinine decreases during continuous RRT and therefore is not a relevant factor for weaning [16]. Studies have shown that an increase in urine output can be used as a marker for RRT weaning [17, 18]. The main limitations of this marker for predicting successful RRT weaning are the potential preservation of urine output during renal failure, the occurrence of urine output before renal recovery, and the negative impact of diuretic use on its predictive ability [17]. Renal function can be assessed not only by urine output but also by the urinary concentration of the waste products of metabolism as measured by urinalysis. We hypothesized that the increase in urine concentration could be a marker of renal function recovery in patients with AKI requiring RRT. Intermittent hemodialysis (IHD) is widely used in RRT weaning because it is less expensive, decreases the risk of catheter infection, and allows early mobilization of the patient, which improves outcomes [19–21]. The aim of the present study was to identify markers in urinalysis for IHD weaning in critically ill patients with AKI.

## Methods

### Setting and study population

We conducted a retrospective single-center cohort study in a ten-bed medical ICU of a university hospital in France. This study was approved by our institutional review board (CPP Sud-Est 6 – IRB00008526 number 2015/CE90) in accordance with French regulations. The board waived the need for signed informed consent for patients included. All consecutive patients with AKI requiring RRT between January 2006 and December 2011 were screened. The inclusion criteria were patients with AKI older than 18 years treated with IHD for at least 7 days and four IHD sessions. The exclusion criteria were patients with a decision to forgo life-sustaining treatment, patients who had undergone renal transplantation, patients treated with continuous RRT at the time of weaning, chronic dialysis patients, and patients with urine output less than 100 ml/24 h before the last dialysis session in the ICU.

### Renal replacement therapy

The criteria for initiation of RRT for AKI were at the discretion of the physician. The determinants usually used for initiation of RRT in our ICU were serum urea concentration greater than 25 mmol/L, serum potassium concentration greater than 6 mmol/L, metabolic acidosis with pH less than 7.15, and acute pulmonary edema due to fluid overload with diuretic resistance. Patients who required RRT were treated with IHD, in some cases preceded by continuous RRT. IHD was promoted when the metabolic disorders were severe. Continuous RRT was promoted when fluid overloading was very high. IHD sessions were performed with the AK 200 Ultra S machine (Gambro, Meyzieu, France) and APS (Asahi PolySulfone) biocompatible dialysis membranes (Hemotech, Ramonville Saint-Agne, France). Anticoagulation was achieved with unfractionated heparin or low molecular weight heparin. Continuous RRT was performed with predilution hemofiltration or postdilution hemodiafiltration (ratio of dialysate to postdilution effluent equal to 1:1). Anticoagulation of continuous RRT was achieved with unfractionated heparin. Vascular access was a temporary double-lumen dialysis catheter positioned in a femoral or internal jugular vein. No protocol was used to make the decision to cease hemodialysis treatment.

### Data collection

Data were collected from consulting patients’ medical records and the electronic databases of the ICU and the Department of Medical Biochemistry at our institution. They comprised baseline demographic characteristics, type of admission, severity of illness and comorbidity score, amine and mechanical ventilation use, baseline serum creatinine, starting and cessation dates of RRT in the ICU, weaning date in the ICU, and diuretic use. Baseline serum creatinine was defined as the lowest serum creatinine value in the year before RRT initiation. Baseline glomerular filtration rate (GFR) was estimated according to the Modified Diet in Renal Disease equation [22]. Severity of illness at ICU admission was assessed on the basis of the Simplified Acute Physiology Score II and Sequential Organ Failure Assessment (SOFA) score. Comorbidities were evaluated by McCabe score. Serum electrolyte, creatinine, and urea concentrations were measured daily at 0700 h. In addition, 24-h urine output values (from 0700 to 0700 h) were collected daily. Concentrations of electrolytes, creatinine, and urea from 24-h urine collections were measured at 0700 h. Daily urinary urea excretion was defined as the product of urine output over 24 h and urinary urea concentration. Urine output was adjusted for the patient’s body weight at ICU admission. A patient was considered weaned in the ICU when dialysis was stopped for at least 1 week. For weaned patients, concentrations of electrolytes, creatinine, and urea in the blood and urine and 24-h urine output values were recorded 2 days after the last dialysis session. This day was chosen because it is the moment at which the decision to stop or to continue IHD is usually made. For unweaned patients, the same data were collected on the day of the last dialysis session in the ICU. One month after ICU discharge, renal outcome was recorded. Renal outcome could be either chronic RRT or renal recovery. Renal recovery was complete if serum creatinine was less than 125 % of baseline creatinine and partial otherwise [23].

### Statistical analysis

Statistical analysis was performed with Stata 13 software (StataCorp, College Station, TX, USA). The tests were two-sided, with a type I error set at α = 0.05. Variables were presented as medians (interquartile range) for continuous data and as the number of patients and associated percentages for categorical parameters. Comparisons between the weaned and unweaned groups were made by using χ^2^ or Fisher’s exact tests for categorical variables and Student’s *t* test or the Mann–Whitney *U* test for quantitative parameters. Assumption of normality was assessed by using the Shapiro–Wilk test, and homoscedasticity was verified by using the Fisher-Snedecor test. To identify independent predictors of IHD weaning, a multivariate logistic regression analysis was performed with IHD weaning as the dependent variable. The interactions between factors were tested. All variables whose values were significantly different between the two groups were included. The predictive ability of urine output, urinary urea concentration, and daily urinary urea excretion was assessed with the area under the receiver operating characteristic curve (AUROC) method. Optimal diagnostic thresholds were determined by Youden’s index [1 − (sensitivity + specificity)]. AUROC curves were compared by using the DeLong method [24]. The sensibility, specificity, and accuracy (percentage of patients correctly classified in the weaned or unweaned groups) were compared by using Cochran’s Q test. Positive and negative predictive values were compared by using Fisher’s exact test.

## Results

### Patient characteristics

**Fig. 1:**
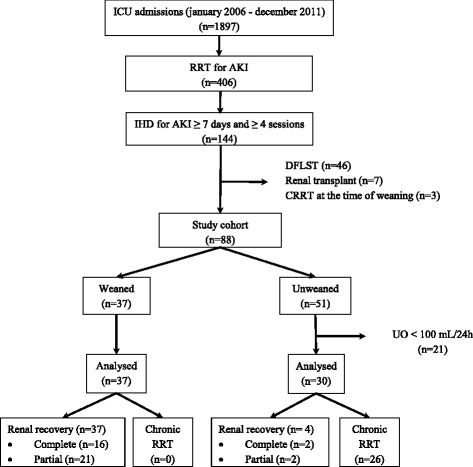
Patient flowchart. *AKI* acute kidney injury, *CRRT* continuous renal replacement therapy, *DFLST* decision to forgo life-sustaining therapy, *IHD* intermittent hemodialysis, *ICU* intensive care unit, *RRT* renal replacement therapy, *UO* urine output

During the 6-year study period, 144 patients treated with IHD for AKI were identified (Fig. 1). Of these, 56 were excluded because of a decision to forgo life-sustaining therapy (*n* = 46) renal transplantation (*n* = 7), or continuous RRT at the time of weaning (*n* = 3). Another 21 were excluded for urine output less than 100 ml/24 h. Thus, 67 patients were finally analyzed, 37 of whom were weaned and 30 of whom were unweaned. Of the 37 weaned patients, none had to restart RRT in the months following RRT weaning. All 30 unweaned patients had dialysis sessions after ICU discharge, but only 4 subsequently recovered renal function and were able to stop RRT a median of 7 (3–8) days after ICU discharge.

The patients’ characteristics are given in Table 1. Weaned patients had chronic kidney failure less frequently and required vasoactive drugs more frequently. In weaned patients, no difference was observed in urine output with or without diuretic use [15.4 (11.2–22.0) vs. 13.3 (9.4–24.2) ml/kg/24 h, *P* = 0.70]. Variables related to RRT during the ICU stay are shown in Table 2.

**Table 1:** Baseline and clinical characteristics of study patients

Variables	All patients (*n* = 67)	Unweaned (*n* = 30)	Weaned (*n* = 37)	*P* value
Age, years	64 (52–73)	63 (47–67)	65 (55–73)	0.22
Male sex, *n* (%)	48 (71.6)	25 (83.3)	23 (62.1)	0.06
Admission type, *n* (%)				0.09
Medical	55 (82.1)	27 (90.0)	28 (75.7)	
Unscheduled surgery	11 (16.4)	2 (6.6)	9 (24.3)	
Scheduled surgery	1 (1.4)	1 (3.3)	0 (0)	
BMI, kg/m^2^	26 (23–31)	25 (21–30)	28 (25–31)	0.22
SOFA score, points	9 (6–13)	9 (6–10)	11 (7–14)	0.07
SAPS2 score, points	61 (45–72)	58 (44–68)	64 (46–73)	0.37
McCabe score, *n* (%)				0.54
0	49 (73.1)	20 (66.7)	29 (78.7)	
1	14 (20.9)	8 (26.7)	6 (16.2)	
2	4 (5.9)	2 (6.7)	2 (5.4)	
Baseline GFR^a^, *n* (%)				<0.001
>90 ml/min/1.73 m^2^	13 (19.4)	2 (6.7)	11 (29.7)	
60–90 ml/min/1.73 m^2^	16 (23.9)	4 (13.3)	12 (32.4)	
30–60 ml/min/1.73 m^2^	16 (23.9)	7 (23.3)	9 (24.3)	
15–30 ml/min/1.73 m^2^	16 (23.9)	11 (36.7)	5 (13.5)	
<15 ml/min/1.73 m^2^	6 (9.0)	6 (20.0)	0 (0.0)	
Contributing factors to AKI, *n* (%)				
Ischemia	60 (89.6)	26 (86.6)	34 (91.8)	0.49
Nephrotoxicity	18 (26.8)	10 (33.3)	8 (24.3)	0.28
Sepsis	42 (62.7)	18 (60.0)	24 (64.8)	0.68
Other	9 (13.4)	4 (13.3)	5 (13.5)	0.98
Length of ICU stay, days	18 (10–31)	11 (7–15)	26 (17–42)	0.01
Renal replacement therapy, days	11 (7–15)	10 (7–15)	11 (8–15)	0.90
Diuretic at the last dialysis session, *n* (%)	7 (10.4)	1 (3.3)	6 (16.2)	0.09
Mechanical ventilation during ICU stay, *n* (%)	46 (68.7)	17 (56.7)	29 (78.3)	0.06
Amines during ICU stay, *n* (%)	47 (70.1)	15 (50.0)	32 (86.5)	0.01

*AKI* acute kidney injury, *BMI* body mass index, *GFR* glomerular filtration rate estimated using Modified Diet in Renal Disease equation, *ICU* intensive care unit, *RRT* renal replacement therapy, *SAPS2* Simplified Acute Physiological Score II, *SOFA* sequential organ failure assessment

Data are presented as median (interquartile range) or count (%)

^a^Baseline GFR classified according to Kidney Disease: Improving Global Outcomes classification

**Table 2:** Variables related to renal replacement therapy during the ICU stay

Variables	All patients (*n* = 67)	Unweaned (*n* = 30)	Weaned (*n* = 37)	*P* value
Duration of RRT, days	11 (7–15)	10 (7–15)	11 (8–15)	0.90
Patients treated with CRRT, *n* (%)	20 (30)	7 (23)	13 (35)	0.05
Duration of CRRT, days	5 (2–7)	3 (3–6.5)	5 (2–7)	0.90
Patients treated with hemofiltration^a^, *n* (%)	9 (13)	3 (10)	6 (16)	0.46
Patients treated with hemodiafiltration^b^, *n* (%)	11 (16)	4 (13)	7 (19)	0.54
Prescribed total effluent flow of CRRT^c^, ml/kg/day	45.7 (44.4–47.0)	42.8 (42.0–43.6)	46.5 (45.3–48.5)	0.23
Blood flow of CRRT, ml/minute	200 (200–200)	200 (200–200)	200 (200–200)	0.34
IHD sessions during ICU stay, *n*	7 (5–10)	7 (5–9)	7 (5–10)	0.47
Duration of IHD session, h	4.1 (3.7–4.6)	4.0 (3.8–4.5)	4.2 (3.5–4.6)	0.88
IHD sessions per week, *n*	5.5 (4.4–6.2)	5.0 (4.4–6.1)	5.8 (4.4–6.4)	0.29
Blood flow of IHD, ml/minute	214 (200–240)	233 (208–250)	209 (200–217)	0.01
Dialysate flow, ml/minute	540 (500–586)	530 (500–571)	545 (500–596)	0.52
K∙t/V/IHD session^d^	0.91 (0.73–1.09)	0.82 (0.71–1.08)	0.95 (0.76–1.09)	0.83
Net ultrafiltration, L/h	0.25 (0.18–0.38)	0.24 (0.18–0.38)	0.27 (0.18–0.38)	0.66
Percentage of IHD sessions with hypotension^e^	16.6 (0.00–25.0)	16.6 (0.00–24.0)	15.5 (0.00–27.7)	0.60

*RRT* renal replacement therapy, *CRRT* continuous renal replacement therapy, *IHD* intermittent hemodialysis

Data are presented as median (interquartile range) or count (%)

^a^All hemofiltration was delivered with predilution mode

^b^All hemodiafiltration was delivered with postdilution mode and a ratio of dialysate to postdilution effluent equal to 1:1

^c^For hemodiafiltration, the total effluent was the sum of the dialysate and the postdilution effluent

^d^K∙t/V was measured with ionic dialysance

^e^Hypotension requiring fluid challenge or increase of vasoactive drug

### Identification of weaning markers

The variables that differed significantly between unweaned and weaned patients were serum creatinine concentration [median 386 (interquartile range 317–468) vs. 253 (191–325) μmol/L, *P* < 0.001], urinary urea concentration [92 (67–123) vs. 160 (124–222) mmol/L, *P* < 0.001], urine osmolality [289 (263–306) vs. 345 (302–375) mmol/L, *P* = 0.01], and urine output [6.5 (4.0–9.2) vs. 14.5 (9.6–22.4) ml/kg/24 h, *P* < 0.001]. No difference was observed in serum urea concentration [20.4 (17.1–25.4) vs. 20.2 (15.9–25.9) mmol/L, *P* = 0.61] and urinary creatinine concentration [4463 (3244–6396) vs. 3922 (2893–6317) μmol/L, *P* = 0.85]. In a multivariate logistic regression model (Table 3), independent predictors for IHD weaning were 24-h urine output [odds ratio 1.59 per ml/kg/24 h increase, 95 % confidence interval (CI) 1.20–2.10, *P* = 0.01] and urinary urea concentration (odds ratio 1.29 per 10 mmol/L increase, 95 % CI 1.01–1.64, *P* = 0.04). These two independent predictors for IHD weaning were also found when the urine output was not adjusted for body weight (Additional file 1).

**Table 3:** Logistic regression model for predicting intermittent hemodialysis weaning

	Univariate analysis			Multivariate analysis^a^		
Parameter	OR	95 % CI	*P* value	OR	95 % CI	*P* value
SCr, per 10 μmol/L	0.96	(0.88–0.96)	<0.001	0.96	(0.90–1.03)	0.27
Uurea, per10 mmol/L	1.27	(1.12–1.44)	<0.001	1.29	(1.01–1.64)	0.04
Uosm, per 10 mmol/L	1.15	(1.04–1.27)	0.01	1.17	(0.94–1.45)	0.16
UO, per ml/kg/24 h	1.33	(1.14–1.55)	0.01	1.59	(1.20–2.10)	0.01

*SCr* serum creatinine, *OR*, odds ratio, *CI* confidence interval, *Uurea* urinary urea concentration, *UO* urine output, *Uosm* urine osmolality

^a^Adjusted for diuretic use

### Predictive value of urine output, urinary urea concentration and daily urea excretion

**Fig. 2:**
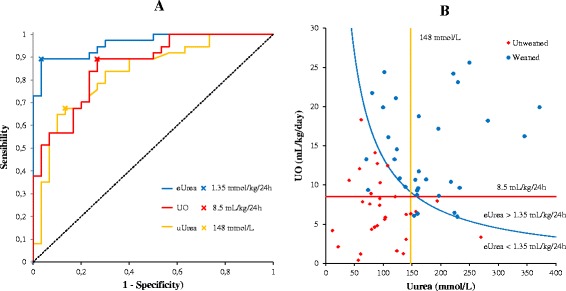
**a** Predictive ability for intermittent hemodialysis weaning. The area under the receiver operating characteristic curve values were 0.86 (0.79–0.94) for UO, 0.83 (0.74–0.92) for uUrea, and 0.96 (0.93–0.99) for eUrea. Thresholds with the highest accuracy are given for each variable. **b** Distribution of weaned and unweaned patients according to UO, uUrea, and eUrea. The *blue curve* corresponds to an eUrea of 1.35 mmol/kg/24 h and was obtained using the following equation: [UO (ml/kg/24 h)/1000] × uUrea (mmol/L) = 1.35 mmol/kg/24 h. *eUrea* daily urinary urea excretion, *uUrea* urinary urea concentration, *UO* urine output

The AUROCs for predicting IHD weaning were 0.86 (95 % CI 0.79–0.94, *P* < 0.001) for urine output, 0.83 (95 % CI 0.74–0.92, *P* < 0.001) for urinary urea concentration, and 0.96 (95 % CI 0.93–0.99, *P* < 0.001) for daily urinary urea excretion (Fig. 2a). The AUROC of daily urinary urea excretion was greater than that of urine output (*P* = 0.02) and urinary urea concentration (*P* = 0.01). The optimal thresholds for IHD weaning were urine output greater than 8.5 ml/kg/24 h, urinary urea concentration greater than 148 mmol/L, and daily urinary urea excretion greater than 1.35 mmol/kg/24 h. The sensitivity, specificity, positive predictive value, negative predictive value, and accuracy for each of these thresholds are shown in Table 4. A daily urinary urea excretion greater than 1.35 mmol/kg/24 h provided better accuracy (92.5 %) than a urine output greater than 8.5 ml/kg/24 h (82.1 %, *P* = 0.03) or a urinary urea concentration greater) than 148 mmol/L (76.1 %, *P* = 0.01) (Table 3). The optimal thresholds for urine output and daily urinary urea excretion unadjusted for the body weight were 826 ml/24 h and 92 mmol/24 h, respectively (Additional file 2). The distribution of weaned and unweaned patients according to values of urine output, urinary urea concentration, and daily urinary urea excretion is shown in Fig. 2b.

**Table 4:** Comparison of diagnostic values

Variables	UO >8.6 ml/kg/24 h	Uurea >148 mmol/L	eUrea >1.35 mmol/kg/24 h	*P* value
Sen, %	89.2^a^	64.9^a^	89.2^b^	0.01
Spe, %	73.3^a^	90.0^b^	96.7^b^	0.01
PPV, %	80.5^a^	88.9^b^	97.1^b^	0.05
NPV, %	84.6	67.5	87.9	0.09
Accuracy, %	82.1^a^	76.1^a^	92.5^b^	0.03

*eUrea* daily urinary urea excretion, *PPV* positive predictive value, *NPV* negative predictive value, *Sen* sensitivity, *Spe* specificity, *UO* urine output, *Uurea* urinary urea concentration

^a,b^Values are significantly different

## Discussion

The main result of this study was the determination of thresholds of urine markers for IHD weaning in ICU patients with AKI. A daily urinary urea excretion greater than 1.35 mmol/kg/24 h was the best marker of renal recovery for IHD weaning.

There is no specific recommendation regarding the criteria for identifying patients who have recovered sufficient renal function to allow RRT weaning. In major randomized controlled trials on RRT [10, 11], the criteria of RRT weaning were based on urine output (greater than 400 ml/24 h), creatinine clearance (greater than 20 ml/minute assessed using 6-h urine collection in patients with urine output higher than 30 ml/h), or the discretion of the physician. In an observational study of 94 patients with postoperative AKI, researchers reported that longer duration of dialysis, age older than 65 years, higher SOFA score, and urine output less than 100 ml/8 h were associated with weaning failure [18]. In their study, the urine output on the day following the last session was significantly greater in weaned patients (1435 ml/24 h vs. 598 ml/24 h). In a post hoc analysis of 529 patients from the BEST study, urine output was the main predictor of successful RRT cessation [17]. In this analysis, the AUROC of patients without diuretics was 0.845 and the best threshold was 436 ml/24 h, but the AUROC decreased to 0.671 with the best threshold at 2330 ml/24 h in patients with diuretics [17]. In our study, we determined one urine output threshold, which was intermediate (8.5 ml/kg/24 h or 826 ml/24 h), because 90 % of the patients received no diuretics. However, the physiological data show that the predictive ability of urine output for recovery of renal function is poor. The two major determinants of urine output are osmotic load, composed mainly of urea and sodium chloride delivery on the distal tubule, and urine concentration. Both these determinants are related to factors other than the recovery of renal function. Osmotic load results from several factors, including GFR, urea generation caused by protein catabolism, and variations in sodium reabsorption in response to circulating blood volume or diuretic use [25]. Urine concentration results from the functional integrity of the renal tubule [26] and the release of arginine vasopressin [27].

Our study shows that daily urinary urea excretion has a better predictive value than urine output or urinary urea concentration alone for IHD weaning in ICU patients with AKI. Daily urinary urea excretion results from the GFR, serum urea concentration, and urea tubular reabsorption. Urea is freely filtered across the glomerulus, and 50 % is reabsorbed in the outer medulla and then secreted in the inner medulla. This recycling of urea creates a concentration gradient in the medulla, which causes the movement of electrolyte and water that leads to urine formation [26]. Consequently, an increase in daily urinary urea excretion requires the recovery of tubular cell integrity and of urine concentration ability. These data suggest that the daily urinary urea excretion is one of the first markers of renal recovery after AKI requiring RRT.

The objective of our study was not to assess the factors that influence the renal recovery. However, we found that most of the weaned patients required vasoactive drugs and that there was a trend toward more continuous RRT in weaned patients than in unweaned patients (Table 2). Furthermore, there was no difference between the patient groups in the percentage of IHD with hypotension requiring fluid challenge or an increase in vasoactive drug use. These data suggest an association between weaned patients and continuous RRT and are consistent with other results that show a beneficial association between continuous RRT and renal recovery [28–30].

Our study has a number of limitations. First, the study design was retrospective. However, data analysis from real clinical practice is the first step in finding new and relevant markers. The collection of data was exhaustive, with multiple screening of patients from several different databases. Hence, the possibility of overlooking a patient meeting the inclusion criteria was very low. Although our data collection was exhaustive and the statistical analysis robust, further prospective studies are needed to assess whether our findings can be used to make clinical decisions. Second, daily urinary urea excretion requires accurate urine collection over 24 h. Urine collection and urinalysis are easily done with urine catheters and are routinely used in clinical practice in the ICU. In addition, 24-h urine collection reduces biases of inaccuracy. Daily urinary urea excretion is a relevant marker for IHD weaning that does not generate additional cost. Third, we assessed weaning from IHD and not from continuous RRT. IHD is widely used for RRT weaning (and is the treatment of choice in our ICU) because it allows early mobilization, which is now a major objective in ICU patients. Daily urinary urea excretion could also be used as a weaning marker for continuous RRT. The measurement of daily urinary urea excretion requires only urine collection and urinalysis and can therefore be performed in ICU patients treated with continuous RRT. Additional studies are needed to assess the threshold of daily urinary urea excretion in continuous RRT. Finally, daily urinary urea excretion is a marker that is related to renal recovery and not the type of RRT.

## Conclusions

To our knowledge, this study is the first to show an association between renal recovery for IHD weaning and both urine output and urinary urea concentration in patients with AKI. Daily urinary urea excretion is better than urine output alone for predicting IHD weaning. A threshold of daily urinary urea excretion greater than 1.35 mmol/kg/24 h was found to be the best marker for weaning ICU patients with AKI from IHD. Additional studies are needed to assess prospectively the role of urinary markers in the strategy of RRT weaning in ICU patients.

## Key messages

- Urine output and urinary urea concentration are both independently related to renal recovery for intermittent hemodialysis weaning.
- Daily urinary urea excretion has a better performance than urine output or urinary urea concentration alone in identifying renal recovery for intermittent hemodialysis weaning.
- Daily urinary urea excretion greater than 1.35 mmol/kg/24 h was found to be the best marker for weaning ICU patients with AKI from intermittent hemodialysis.

## Additional files

Additional file 1: Table S1. Logistic regression model for predicting intermittent hemodialysis weaning with variables not adjusted for body weight. (DOCX 19 kb) Additional file 2: Table S2. Comparison of diagnostic values with variables not adjusted for body weight. (DOCX 20 kb)

## Abbreviations

AKI: acute kidney injury
AUROC: area under the receiver operating characteristic curve
BMI: body mass index
CI: confidence interval
DFLST: decision to forgo life-sustaining therapy
eUrea: daily urinary urea excretion
GFR: glomerular filtration rate estimated using Modified Diet in Renal Disease equation
ICU: intensive care unit
IHD: intermittent hemodialysis
NPV: negative predictive value
OR: odds ratio
PPV: positive predictive value
RRT: renal replacement therapy
SAPS2: Simplified Acute Physiology Score II
SCr: serum creatinine
Sen: sensitivity
SOFA: Sequential Organ Failure Assessment
Spe: specificity
UO: urine output
Uosm: urine osmolality
Uurea: urinary urea concentration
