# Supplementary material for: Daily urinary urea excretion to guide intermittent hemodialysis weaning in critically ill patients
Source: Crit Care. 2016 Feb 19;20:43. doi: 10.1186/s13054-016-1225-5 (PMC4761179; doi:10.1186/s13054-016-1225-5)
Supplement: Additional file 1: Table S1. — Logistic regression model for predicting intermittent hemodialysis weaning with variables not adjusted for body weight. (DOCX 19 kb) [file 13054_2016_1225_MOESM1_ESM.docx]

**Table S1.** Logistic regression model for predicting intermittent hemodialysis weaning with variables non-adjusted for body weight.

|  | **Univariate analysis** | | | **Multivariate analysis*** | | |
| --- | --- | --- | --- | --- | --- | --- |
| **Parameter** | **OR** | **CI 95%** | ***P* value** | **OR** | **CI 95%** | ***P* value** |
| sCreatinine, per 10 µmol/L | 0.92 | (0.88 – 0.96) | <0.001 | 0.92 | (0.85 – 1.01) | 0.07 |
| uUrea, per10 mmol/L | 1.27 | (1.12 – 1.44) | <0.001 | 1.37 | (1.01 – 1.85) | 0.04 |
| uOsm, per 10 mmol/L | 1.15 | (1.04 – 1.27) | 0.01 | 1.26 | (0.94 – 1.70) | 0.13 |
| UO, per 100 mL/day | 1.64 | (1.28 – 2.10) | <0.001 | 3.39 | (1.53 – 7.51) | 0.01 |

sCreatinine, serum creatinine; OR, odds ratio; CI 95%, Confidence interval 95%; uUrea, urinary urea concentration; UO, urine output; uOsm, urine osmolality

* adjusted for diuretic use
