# Supplementary material for: Daily urinary urea excretion to guide intermittent hemodialysis weaning in critically ill patients
Source: Crit Care. 2016 Feb 19;20:43. doi: 10.1186/s13054-016-1225-5 (PMC4761179; doi:10.1186/s13054-016-1225-5)
Supplement: Additional file 2: Table S2. — Comparison of diagnostic values with variables not adjusted for body weight. (DOCX 20 kb) [file 13054_2016_1225_MOESM2_ESM.docx]

**Table S2.** Comparison of diagnostic values with variables non-adjusted for body weight.

| **Variables** | **UO**  **> 826 mL/24h** | **uUrea**  **> 148 mmol/L** | **eUrea**  **> 92 mmol/24h** | ***P* value** |
| --- | --- | --- | --- | --- |
| Sen % | 73.0^a^ | 64.9^a^ | 89.2^b^ | 0.04 |
| Spe % | 90.0 | 90.0 | 96.7 | 0.51 |
| PPV % | 90.0 | 88.9 | 97.1 | 0.37 |
| NPV % | 73.0 | 67.5 | 87.9 | 0.11 |
| Accuracy % | 80.6^a^ | 76.1^a^ | 92.5^b^ | 0.02 |

eUrea, daily urinary urea excretion; PPV, positive predictive value; NPV, negative predictive value; Sen, sensitivity; Spe, specificity; UO, urine output; uUrea, urinary urea concentration. Values with different letters are significantly different.
